# A Multisensor Device Intended as an IoT Element for Indoor Environment Monitoring

**DOI:** 10.3390/s24051461

**Published:** 2024-02-23

**Authors:** Andrzej Szczurek, Dawid Gonstał, Monika Maciejewska

**Affiliations:** 1 Faculty of Environmental Engineering, Wroclaw University of Science and Technology, Wybrzeże Wyspiańskiego 27, 50-370 Wrocław, Poland; andrzej.szczurek@pwr.edu.pl; 2 Independent Researcher, ul. Cukrowa 9/11, 52-316 Wrocław, Poland; dawid.gonstal@gmail.com

**Keywords:** indoor environmental quality, indoor air quality, sensor, monitoring, internet of things

## Abstract

This work presents a multisensor device which is intended as an element of IoT for indoor environment (IE) monitoring. It is a portable, small-size, lightweight, energy-efficient direct-reading instrument. The device has an innovative design and construction. It offers real-time measurements of a wide spectrum of physical and chemical quantities (light intensity, temperature, relative humidity, pressure, CO_2_ concentration, content of volatile organic compounds including formaldehyde, NO_2_, and particulate matter), data storage (microSD; server as an option), transmission (WiFi; GSM and Ethernet as options), and visualization (smartphone application; PC as an option). Commercial low-cost sensors were utilized, which have been arranged in the individual sensing modules. In the case of gas sensors, dynamic exposure was chosen to ensure a minimum response time. The MQTT protocol was applied for data transmission and communication with other devices, as well as with the user. The multisensor device can collect huge amounts of data about the indoor environment to provide the respective information to the IoT. The device can be configured to control actuators of various auxiliary devices and equipment including external systems used for ventilation, heating, and air conditioning. The prototype is fully operational. The exemplary results of IE monitoring were shown.

## 1. Introduction

People spend the majority of their time indoors. The quality of the indoor environment (IEQ) has a strong impact on human health, safety, productivity, and overall well-being [1,2,3]. There is an increasing amount of scientific evidence indicating that a range of health problems and complaints are associated with poor IEQ. They range from temporary sensory irritation of the respiratory tract to diseases that can be life-threatening. Indoor environmental quality is most simply described as the conditions inside of a building. This term includes air quality, thermal comfort, moisture, visual and acoustic conditions, daylight, lighting, and electromagnetic radiation levels [4]. A particularly important element of IEQ is the indoor air quality (IAQ). This refers to the air quality within and around buildings and structures, especially as it relates to the health and comfort of building occupants.

Recently, there has been an increased demand for improving IEQ. Different strategies are proposed [5]. The internet of things (IoT) may play an important role in attaining high IEQ. IoT allows the connection of various physical objects to the Internet, enabling them to be monitored and controlled, while the relevant data are collected in real time. IoT offers numerous benefits, such as improving energy efficiency, security, convenience, and the quality of residents’ lives. Moreover, the improvement of IEQ based on IoT may be attained. However, the appropriate information is needed about physical, chemical, and biological conditions inside buildings.

Generally speaking, many methods and techniques are available to provide the necessary data. One of them is based on the observation of the individual phenomena, elements, or features of the indoor environment. This approach presents advantages but also serious shortcomings; e.g., not everything is noticeable, observation needs to be long-lasting so it is time-consuming, it is costly, and the personal bias of the observer affects the information in many ways [6,7]. In addition, the multiplicity of factors influencing IEQ often makes observations inaccurate, incapable of capturing the complexity of situations, and inadequate for practical applications.

Another strategy relies on measurements of indoor microclimate parameters that express the physical, chemical, and biological conditions experienced by residents and workers. The microclimate in buildings is represented by environmental variables including temperature, humidity, airflow (thermal microclimate), ionizing and non-ionizing radiation, noise, vibration, and chemical and biological contaminants [8].

IEQ monitoring is challenging due to the complexity of the indoor microclimate. A range of biotic and abiotic factors can affect conditions to which occupants are exposed. In addition, they present temporal and spatial variation. Hence, a specific set of measurement methods and tools is required to obtain information about indoor parameters. The methods cannot be expensive or time-consuming, and they shall not require trained and experienced personnel. The provided information has to be objective and quantifiable. It is worth mentioning that less accurate but simpler, quicker, and more convenient methods are entirely satisfactory and desirable for routine monitoring [9]. Various techniques may be used for the measurements of parameters of the indoor environment [10]. A very effective approach is based on sensor technology. Nowadays, sensing technology receives increased attention in the field of indoor environment monitoring, because it is unique in its ability to record various temporal data, in real time [11]. However, it has to be underlined that achieving high-quality IEQ monitoring with inexpensive sensors is challenging.

Typically, microclimate monitoring inside a building is based on periodic or continuous measurements. Most of the existing systems for continuous, real-time monitoring of indoor environments are stationary wire or wireless sensor networks [12]. A sensor network is a group of sensors distributed in numerous measurement points that collect and, ultimately, send the data to a central location for storage, viewing, and analysis. It can collect a huge amount of data about the environment to quickly assess conditions inside a building and immediately actuate controls to attain adequate correction. The sensor networks provide the most detailed information about the current state of the indoor microclimate, as well as the temporal variation of IEQ [13]. Until recently, the strategy based on continuous measurements was discouraged by the cost of the monitoring networks and the efforts required to install and calibrate sensors. Fitting this equipment in the existing building structures, but also in new buildings, is a costly endeavor. At present, a limited number of stationary systems for the continuous monitoring of IEQ is available.

Since sensor networks are often prohibitively expensive and impractical, especially for large-scale applications or with the fine-grained deployment of sensors, the information about the indoor microclimate is most willingly acquired using portable direct-reading measurement instruments [14]. There are many types of these devices. The available instruments vary strongly in complexity, price, and performance characteristics [15]. The progress in electronics and sensor technology allows us to significantly improve the direct-reading instruments and enhance their application potential.

This paper aims to present an innovative design and construction of a multisensor device that operates in situ and provides data characterizing IEQ. The proposed solution realizes the sensors’ exposure upon dynamic gas sampling. It is distinctive since the passive sampling strategy is commonly used in direct-reading devices developed with IAQ and IEQ monitoring applications in mind. The novelty lies in the technical aspect of the presented solution. The especially interesting element in the device is the original arrangement of the sensor chamber, which influences the sensor signal dynamics. It also allows the application of a fan instead of a vacuum pump to draw the gas sample, which reduces the device’s energy consumption. In our opinion, the presented multisensing device may be a valuable option in indoor environment monitoring applications. It can be used as a portable instrument or an element (node) of a monitoring network.

## 2. Assumptions

It was assumed that the developed sensor device should be based on sensor technology. Nowadays, sensors receive increased attention in the field of indoor environment monitoring, because they offer the possibility to construct automated devices that:

- Can work over extended periods (weeks or months) with minimum operator attention/intervention;
- Are widely accessible;
- Provide continuous, online measurements of various physical and chemical quantities with a high temporal resolution;
- Are simple to use;
- Offer good accuracy, sensitivity, and repeatability of measurements.

Sensors use different detection principles and, therefore, they vary strongly in complexity, price, and performance characteristics.

Factors like cost, power consumption, and space utilization were considered for the design of the presented device. It was assumed that it has to be accepted by the occupants as an integral part of an indoor environment. The device cannot obstruct the occupants’ activity in the room. It should (1) occupy little space and not disrupt the spatial organization inside the room, (2) be noiseless (within achievable limits), (3) not affect the composition of air and its natural circulation in the room, and (4) not affect thermal conditions (it cannot release or consume significant amounts of heat).

Due to the complexity of factors influencing the indoor microclimate, the device was designed for the measurement of numerous environmental parameters. Its measurement potential was targeted at temperature, relative humidity, pressure, CO_2_ concentration, content of volatile organic compounds including formaldehyde, concentration of NOx, and concentration of particulate matter, and light intensity. Temporal variations of IEQ cause continuous measurements to be required. It was assumed that, depending on the needs, the monitoring sessions can be short- or long-term, but data are always provided in real time. Due to the spatial diversity of indoor parameters, the device was designed as a portable construction. The location of this instrument may be quickly and easily changed within one room, as well as between rooms. The actual location of the device may be chosen based on room arrangement and the availability of technical infrastructure (power supply and WiFi). The indoor space is usually limited; therefore, the device was designed as a compact, robust construction, made of durable elements. The important requirement was its portability and the possibility of application in IoT.

## 3. Multisensor Device

**Figure 1:**
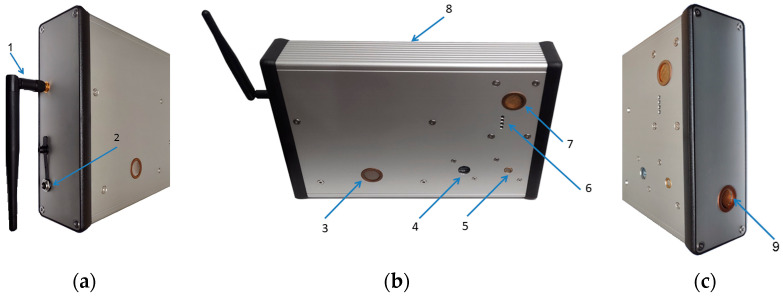
The measurement device (220 mm × 165 mm × 51.5 mm, weight 1300 g). (**a**) Left side view. (**b**) Front view. (**c**) Right side view. 1—WiFi antenna, 2—power socket DC 5 V, 3—gas inlet for multi-cell sensing module, 4—light intensity sensor, 5—pressure sensor, 6—gas outlet for PM5003 sensor module, 7—gas inlet for the PM5003 sensor module, 8—aluminum housing, and 9—gas outlet for multi-cell sensing module.

A general view of the measurement device is shown in Figure 1.

Three issues are critical in characterizing the device: the operating mode (see Section 3.1), the operations and functional modules implemented (see Section 3.2), and the configuration and structure of the measurement system (see Section 3.3).

### 3.1. Operating Mode

The device is intended for in situ measurements. The operating mode is based on continuous, short- or long-term measurements. The placement of the device during the measurement session is fixed but can be easily changed.

The instrument was designed as the sample flow system. In this operating mode, sensors are placed in the gas stream, which enables the rapid exchange of atmosphere inside the measurement chamber. The direct-reading instruments with the sample flow are convenient because the measurement process is short and easy to handle.

### 3.2. Operations and Functional Modules Implemented

Eight functions are fundamental to sensor device operation:

- Gas sampling;
- The generation of sensor responses to physical and chemical quantities;
- The generation of an electrical measurement signal;
- Electrical signal conditioning;
- Electrical signal conversion;
- Data acquisition and storage;
- Signal preprocessing;
- Data visualization;
- Calibration.

#### 3.2.1. Gas Sampling

The operation of direct-reading instruments may be based on a passive (diffusive) or active (dynamic) sampling method.

Passive sampling is simple and soundless, and requires zero energy consumption. The sampling module is very small, which simplifies the construction of the sensing device. The volume of the gas sample is small; thus, the sampling operation has little effect on the composition of indoor air around the device. However, the passive sampling also has shortcomings. The drawback of this approach is that, when measurements are carried out, sensors are directly exposed to variations in environmental conditions. Local air turbulence causes the sensors’ signals to fluctuate and contain noisy, transient patterns. This may be overcome by averaging. However, our application is meant to be sensitive to short-term events occurring in indoor environments; therefore, the device providing time-integrated data is not attractive. Passive sampling does not allow for a quick response of the sensor to changes in indoor air composition. This results from the limited dynamics of diffusion. The diffusive sampling requires direct contact between the sensor and the surrounding gas. Hence, the result of measurements based on the passive principle represents the gas composition in the area of a few cubic centimeters around the sensor. The air exchange rate induced by diffusion is low. Hence, the sensor response to the surrounding gas may be unrepresentative for more distant points. It is known that the properties of the indoor environment usually show high spatial and temporal variability.

Active sampling partially reduced the shortcomings of the passive strategy. In this approach, indoor air is continuously withdrawn from the measurement location and then transported towards the sensor. The dynamics of the sensor response upon active sampling is much higher compared to when passive sampling is used. The spatial representativeness of the obtained measurement result is better since it refers to a larger space. Of course, dynamic sampling also has several limitations. They result from the necessity of using a gas mover, sensor chamber, and system for gas delivery. These requirements affect the weight, size, and compactness of the sensing device, as well as its energy consumption. This work aimed to solve these problems. The novelty of this work lies in the technical aspect of the proposed solution, which realizes sensor exposure upon dynamic sampling. In our opinion, the developed design of the sensing device is worth presenting. The especially interesting element is the original arrangement of the sensor chamber, which influences the signal dynamics and allows the application of a fan instead of a pump to draw the gas sample, hence reducing the device’s energy consumption. In our opinion, the proposed approach may be a valuable option in indoor environment monitoring applications. Of course, the presented version of the device is not the ultimate one. It can and will be modified based on the accumulated measurement experience. The prototype device we developed became operational several weeks ago, and we just started the measurements to test its performance.

#### 3.2.2. Generation of Sensor Responses to Physical and Chemical Quantities

The measurement of physical and chemical quantities in the device is based on sensor technology. The environmental stimulus causes changes in a defined electrical parameter. The magnitude of change is a function of the value of a measured quantity. The resulting sensor response produces an analog output that is dependent on the quantity being monitored.

#### 3.2.3. Generation of an Electrical Measurement Signal

The sensor response has to be transformed into an electrical signal (voltage change).

A signal is the main source of information in the presented measurement method. Its generation is a key issue in the sensor device operation. The signal generation is performed using interface circuits. In practice, the sensor response to the measured quantity was recorded on a load resistor. An output signal was generated in the form of a sequence of raw voltage variations on the load resistance connected to the sensor.

**Figure 2:**
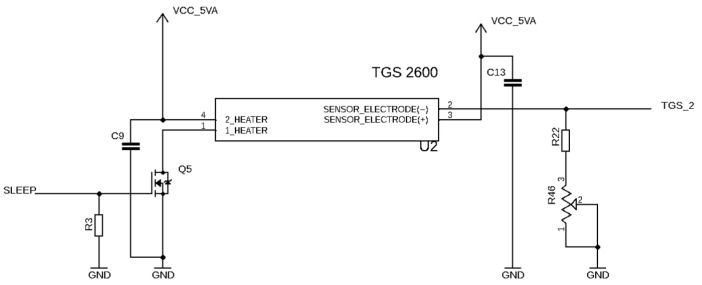
The voltage divider for sensor TGS2600. It is identical to the one applied for sensor TGS2602. Numbers 1–4 indicate pins, in accordance with sensor data sheet.

In the presented device, the signal was the voltage at the output of the voltage divider; see Figure 2. The voltage divider construction is based on the product information offered for sensors TGS2600 and TGS2602. The applied load resistance was between 5 kOhm and 15 kOhm. Its value is regulated by the rotary potentiometer (0–10 kOhm). The minimum load resistance (5 kOhm) was determined by resistors R13 and R14.

#### 3.2.4. Electrical Signal Conditioning

The electrical signals generated by sensor interface circuits are often not adequate for acquisition and must be further processed by several analog signal conditioning circuits. Conditioners standardize the level of the signal from the measurement transducer to the range of the input voltage of the analog-to-digital converter (ADC). The four basic roles of these circuits are buffering, amplification, filtering, compensation, and some other special functions. Signals may be contaminated with diverse noises.

**Figure 3:**
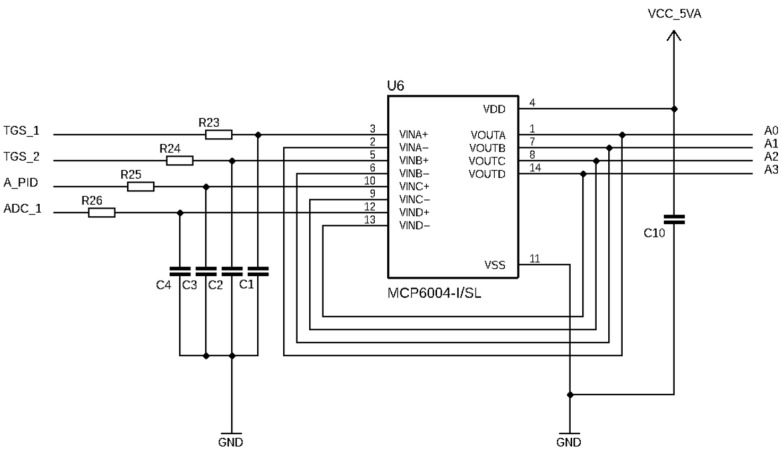
Electronic diagram of an active RC filter applied in the device. Numbers 1–14 indicate pins, in accordance with MCP6004 data sheet.

The appropriate active RC filters based on the MCP6004 (Microchip Technology, Chandler, AZ, USA) operational amplifier were used to provide maximum noise reduction, as shown in Figure 3. The applied values were R = 100 kOhm and C = 1 μF to attain a cut-off frequency of 1.59 Hz.

#### 3.2.5. Electrical Signal Conversion

The measurement signal at the output of a converter is an analog quantity, described by a continuous function x(y), usually a time-dependent variable x(t). To process measurement signals in digital systems, analog signals must be converted into digital signals, which are recorded as binary words. This conversion is performed by analog-to-digital converters. The reverse operation, that is, digital-to-analog (D/A) conversion, is very often necessary as well. This operation is performed by digital-to-analog converters (DACs).

The dedicated electronic system ADS1115 (Texas Instruments Inc, Dallas, TX, USA) was used to produce a digital signal that a computer can read. The sampling rate, a parameter describing the reduction of a continuous-time signal to a discrete-time signal, was in this study much higher than variations of sensor responses. The applied ADC converter allows us to perform between 8 and 860 conversions during 1 s. The digital signal exhaustively reflected the changes in the sensor resistivity caused by the variations in the measured quantity. The key parameter of ADC converters is the resolution. This parameter is a discrete value indicating the accuracy of signal quantization, which represents the voltage change at the output of the active RC filters. The applied ADS1115 was adjusted to work with the 5 V voltage at the resolution of 12 bits, which allows for voltage signal quantization with an accuracy of up to 1.22 mV. Based on our experience, this resolution is sufficient for detecting the changes in the monitored environment, provided the gas sensor measurement time resolution is at the level of seconds. Using a 12-bit resolution, we also save on the size of the data transfer, its time, and frequency. The applied ADS1115 is widely available and cheap.

#### 3.2.6. Data Acquisition and Storage

An acquisition module is used to convert the data into an interpretable format, and then to collect it. In the device, the data collection is realized in real time, at regular default intervals of 10 s. This parameter is determined by the optimized algorithm of the individual sensor readout. The applied sequence is as follows:

Start the UART configuration for the PMS5003 sensor;
PMS5003 sensor—start the measurement process;
Start the UART configuration for the CO_2_ sensor Cozir CO_2_ Blink;
Cozir CO_2_ Blink sensor—Power ON and start initialization (time needed < 4 s);
Read date and time from RTC (real-time clock);
Read from BH1750 sensor;
Read data from ADC converters (TGS2600, TGS2602, PID-AH2);
Read from Grove Multichannel Gas Sensor v2 sensor;
Read from MPL3115A2 sensor;
Read from Cozir CO_2_ Blink sensor;
Cozir CO_2_ Blink sensor—Power OFF;
Start the UART configuration for the PMS5003 sensor;
Read from PMS5003 sensor;
Save data on the microSD card.

The measurement data are stored electronically on an SD card, together with parameters representing the actual date and time. The high-accuracy real-time clock (RTC) DS3231 (Analog Devices, Wilmington, CA, USA) was applied to provide the time in seconds, minutes, hours, dates, months, and years. The clock is fitted with an alternate source of power (battery), so it would continue to keep time even when the primary source becomes unavailable and the device configuration does not have to be performed again. The data are recorded continuously upon fragmentation to form the 200 kB files. This solution allows for the minimization of the network load and guarantees the maintenance of a stable connection. All data collected by the device are available remotely, in real time. Before the data are stored on the memory card, they are recorded in the global table ”SensData_arr[i]”, which stores 21 values in the float format. Both active threads may read the table.

#### 3.2.7. Signal Preprocessing

The sampled signal is usually digitally preprocessed. Although signal preprocessing depends on the application, a series of steps are commonly carried out. Three general steps can be used in preprocessing techniques: baseline manipulation, compression, and normalization. Signal processing is carried out digitally on the microcontroller or carried out externally by a computer. The use of separate signal-processing hardware increases the costs and affects the portability of the instrument due to the increase in size, weight, and power consumption. A good solution to this problem is the integration of the sensor and the signal-processing circuit on a single chip or in a single package. In the presented device, we focused on collecting raw measurement data and no signal preprocessing was involved.

#### 3.2.8. Data Visualization

**Figure 4:**
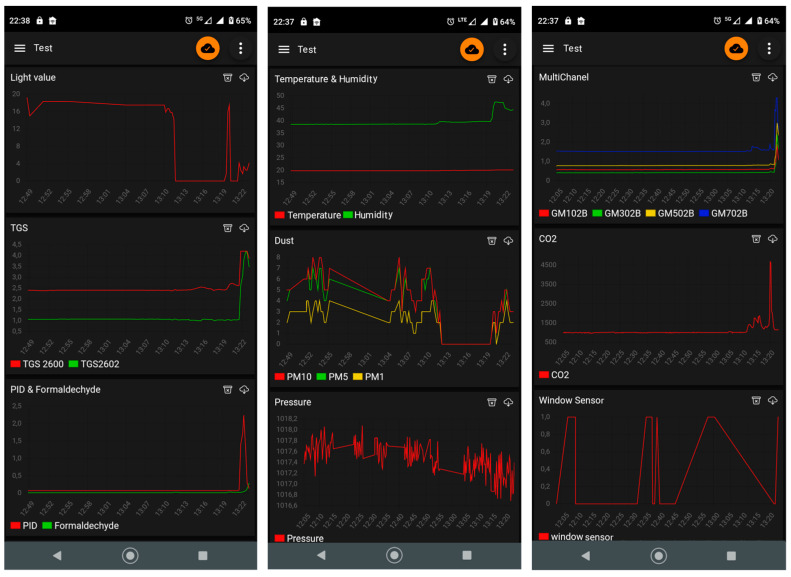
Real-time visualization of the data collected by the measurement device utilizing the “IoT MQTT Panel” application.

The data collected by the device are presented on the controller PC/laptop or smartphone in real time. The graphical display enables the user to identify trends and patterns in the measured parameters of the indoor environment. Figure 4 presents the real-time visualization of the data collected by the presented device. The application “IoT MQTT Panel” was utilized. It has been installed on a smartphone with the Android operation system.

#### 3.2.9. Calibration

The problem of sensor device calibration is complex. It has to be considered from two perspectives: the individual sensors and the sensing device. Sensors usually have to be calibrated at the device level when used as a part of the measurement system.

Depending on the type of information the sensing device is expected to provide, its calibration can be quite easy, or complicated and expensive. The device we present may provide quantitative or qualitative information. The first one is represented by the measured values of physical and chemical indicators of the indoor environment, e.g., temperature, relative humidity, carbon dioxide concentration, etc. The device may also be used as a source of qualitative information, which is expressed in terms of the defined categories representing conditions/situations taking place in the indoor environment. The particular responses of different sensors are assigned to various categories of conditions/situations observed in the environment. This approach has certain limitations, but it is realistic and may turn out to be more practical than focusing on the results of the measurements of the individual parameters of the indoor environment.

##### Calibration of the Sensing Device for Quantitative Information

Calibration can be understood as the comparison of measurement values delivered by a device under test with those of a calibration standard of known accuracy. The term calibration is also used to refer to the process of adjusting the raw sensor readings to obtain corrected values. It is assumed that sensors are calibrated in the factory, during the production stage. However, environmental and operational conditions, as well as improper maintenance, can degrade the measurement performance, resulting in faulty measurement data. Recalibration is usually required to ensure the proper operation of a measurement device. A device cannot be calibrated without being compared against a known reference. Hence, adjusting the reading of a device is only a correction, not a calibration. Recalibration can be performed in accredited laboratories, or by calibration services, usually under controlled conditions.

- Physical sensor calibration

It was assumed, that physical sensors are calibrated by manufacturers. The validity of the physical sensor calibration is usually long. Hence, recalibration was not taken into account.

- Chemical sensor calibration

Gas sensor calibration has to take place in a controlled environment. Typically, a calibration procedure is carried out by placing the sensors inside a chamber where the concentration level of the target gas is kept at a known level. The calibration of the gas sensor involves two steps: First, the “zero” must be set, and, then, the “span” must be calibrated. In practice, most calibration gases are purchased from commercial suppliers. Bottled nitrogen or synthetic air is a good method to establish the “zero”. The span calibration is dependent on the gas type and its concentration range. Methods of span calibration include:

- Premixed calibration;
- A calibrator with constant temperature, flow regulation, and gas permeation devices;
- The use of gas mixing;
- The use of calibrating liquid chemical mixtures.

When it is not possible to use such methods, sensor parameters can be adjusted with reference to another measurement device.

The traditional calibration process is both time-consuming and costly. It shall be performed in a controlled environment. Sensors shall not be calibrated in uncontrolled conditions, because poor or incomplete calibration can lead to significant measurement errors. A good option is to send sensors to the specialized service. Our sensing device features a modular design, with each sensor placed in its compartment in the gas sensor module. The sensors’ replacement in this design is easy.

##### Calibration of the Sensing Device for Qualitative Information

The calibration of the sensing device for qualitative information is an alternative to the calibration carried out in tightly controlled scenarios (e.g., inside chambers), where the sensors are exposed to constant concentration levels of particular species for a predefined time. In this strategy, sensor responses are measured in environmental conditions that represent a given category of the environmental conditions/situation. The obtained data are used as a reference. This “pattern” is placed in the library of the sensing device. The calibration of the sensing device for qualitative information can be performed easily by the device user. This gives a more realistic representation of the particular environment because it will be representative of various conditions that may occur there. We intend to realize this approach in our future work.

### 3.3. Configuration and Structure of the Measurement System

The sensor device has a modular construction. It consists of the following components:

- The multi-cell sensing module;
- The sensing module PMS5003;
- Sensors for the measurement of light intensity and pressure;
- Filters;
- The pneumatic assembly;
- Interfaces and data transmission;
- The microcontroller;
- The power supply;
- The instrument housing.

#### 3.3.1. Multi-Cell Sensing Module

**Figure 5:**
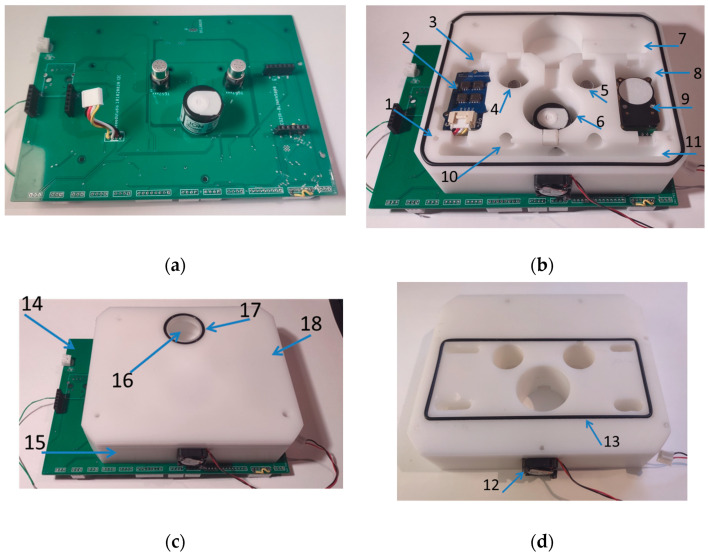
Multi-cell sensing module. (**a**) PCB fitted with TGS2600, TGS2602, and PID-AH2 sensors. (**b**) Measurement chamber made of PTFE without closing top plate; top view. Sensors inside measurement cells are shown. (**c**) Measurement chamber covered with the top plate; top view. (**d**) Measurement chamber; bottom view. 1—thread for the mounting screw, 2—Grove Multichannel Gas Sensor V2 inside sensor cell, 3—gas inlet channel (4×, one for each sensor cell), 4—TGS2600 inside sensor cell, 5—TGS2602 inside sensor cell, 6—PID-AH2 inside sensor cell, 7—gas inlet channel, 8—sealing between the PTFE chamber and the closing top plate, 9—Cozir-Blink5000 inside sensor cell, 10—outlet gas channel (4×, one for each sensor cell), 11—outlet gas collector, 12—fan, 13—sealing between the PTFE chamber and the PCB, 14—PCB, 15—PTFE measurement chamber with sensor cells inside, 16—gas inlet, 17—sealing between the multi-cell sensing module and the device housing, and 18—closing top plate.

The multi-cell sensing module is shown in Figure 5.

It is a small electronic unit that integrates different sensors, transducers, and signal-processing devices into a handy circuit card (system) that is easy to insert into the measurement instrument. It is characterized by a compact design. Its size is 128 mm × 108 mm × 30 mm. The elements of this module are lightweight, low-cost, and robust enough to be successful in its use. The multi-cell sensing module consists of:

- The printed circuit board (PCB);
- Sensors;
- The measurement chamber.

**Figure 6:**
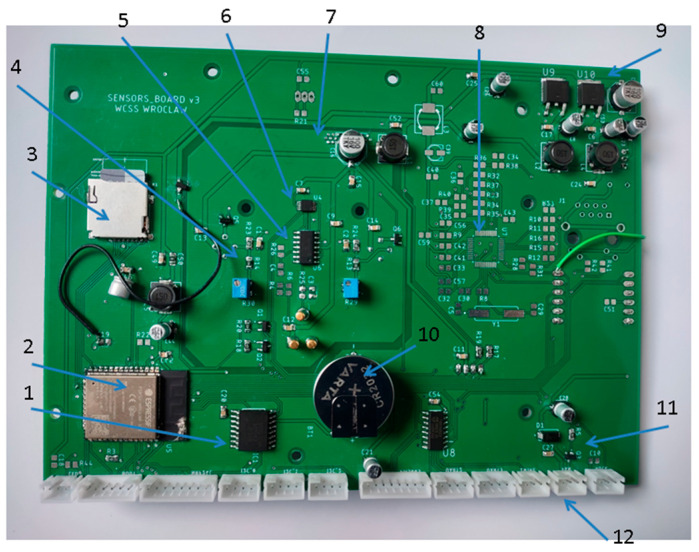
Printed circuit board (PCB). The bottom of the PCB is shown, including multiple electronic elements and circuits, which control the operation of the entire device. 1—RTC clock, 2—microcontroller ESP32, 3—microSD card, 4—voltage divider for TGS2600 and TGS2602, 5—active RC filters, 6 —ADC converter, 7—separated section of analogue electronics, 8—optional Ethernet communication module, 9—power supply circuit, 10—alternate battery for RTC clock, 11—output power for DAC converter which controls the fan, and 12—communication interfaces 4x UART, 3x I2C, 1x SPI, 1x 1-Wire.

The PCB constitutes the basis of the multi-cell sensing module; see Figure 5a,b. It is connected to the power supply and fitted with multiple electronic elements and circuits, which control the operation of the entire measurement device, as shown in Figure 6. They are located on the bottom side of the PCB. The PCB is also fitted with connectors for sensors. They are mounted on the top side of the PCB; see Figure 5a. The sensors sit in the dedicated sockets which allow for multiple exchanges of the sensing elements without soldering; see Figure 5a.

The multi-cell sensing module was designed to measure chemical quantities, which include carbon dioxide (CO_2_), hydrogen (H_2_), volatile organic compounds (VOCs, carbon monoxide (CO), nitrogen dioxide (NO_2_), ethanol (C_2_H_5_OH), and isobutylene((CH_3_)_2_C=CH_2_)). It is fitted with the following sensors:

- TGS2600;
- TGS2602;
- PID-AH2;
- Cozir-Blink5000;
- Grove Multichannel Gas Sensor V2.

The measurement characteristics of the applied sensors are provided in Table 1.

The sensors used in the platform allow for a rapid, continuous, online detection of different volatile substances. They show good accuracy and sensitivity; however, they present partial or overlapping sensitivities. Except for the PID-AH-2 sensor, the applied sensing elements are not bulky in size or heavy in weight. Their power consumption is relatively small. These devices are widely available, affordable, and durable. They can be easily integrated into a multiparameter measurement system.

From a measurement point of view, the chemical air quality is a challenging aspect of the indoor environment. Its complexity is associated with the presence of versatile VOCs at various concentrations in indoor air. For this reason, several sensors responding to VOCs were included in the device. They represent various measurement principles and sensitivities, offering to build an exhaustive representation of indoor air quality in sensing device responses. By all means, the sensor selection is not ultimate. The modular construction of the device allows for easy replacements and expansion. Special attention is paid to MEMS sensors due to their small size and low energy consumption.

Sensors were located in the specially designed measurement chamber, which was made of politetrafluoroethylene (PTFE); see Figure 5. The measurement chamber sits on top of the PCB (see Figure 5b) and the joint between them is airtight (see Figure 5d). The chamber is covered with the closing top plate and this joint is airtight as well (see Figure 5b,c) The measurement chamber comprises flow-through compartments with very small internal volumes, as shown in Table 2. The total free volume inside the chamber is 72.7 cm^3^.

**Figure 7:**
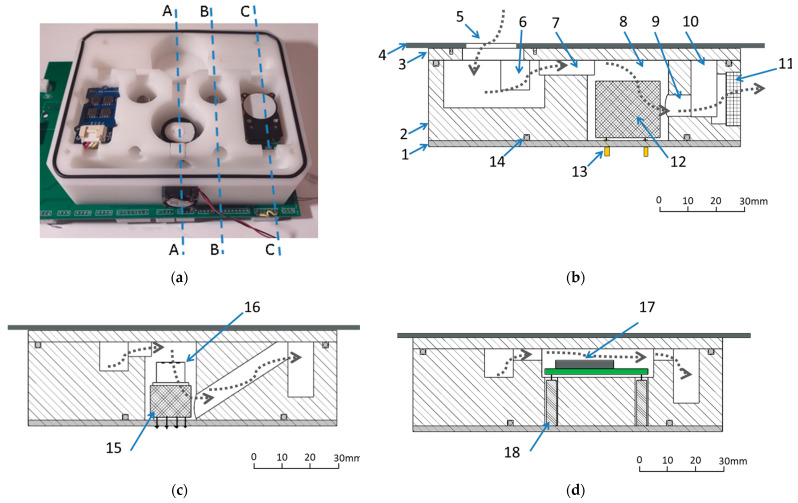
Gas flow inside the measurement chamber The gas flow is indicated using the dashed line. (**a**) Measurement chamber with cross-sections indicated. (**b**) Cross-section A-A, through the PID-AH2 sensor cell. (**c**) Cross-section B-B, through the TGS2602 sensor cell (identical as for TGS2600). (**d**) Cross-section C-C, through the Cozir-Blink5000 (identical as for Grove Multichannel Gas Sensor V2 sensor cell). 1—PCB, 2—measurement chamber PTFE corps, 3—closing top plate, 4—housing of the measurement device, 5—gas inlet through the filter, 6—inlet collector, 7—inlet channel, 8—measurement cell, 9—outlet channel, 10—outlet collector, 11—fan, 12—PID-AH2 sensor, 13—PID AH2 socket, 14—O-ring sealing (3×), 15—TGS2602 sensor, 16—TGS2602 socket, 17—Cozir-Blink5000 sensor, and 18—Cozir-Blink5000 socket.

The dynamics of fluid were taken into consideration when designing the sensor chamber to guarantee homogeneous flow conditions and small gas velocity gradients. The sensor position in a measurement chamber may affect its response. Hence, the key point in designing the related geometry is to ensure that the injected gas sample reaches steady and uniform conditions within the chamber in a time as short as possible, while minimizing the presence of stagnant and/or recirculating regions and, therefore, maximizing the contact of the sensors’ surface with the test gas. As shown in Figure 7, the measurement chamber is fitted with a gas inlet and outlet. Inside the chamber, the gas flows through an internal system of channels, 7 mm in diameter, which connect the individual compartments with the gas inlet and gas outlet. The gas flows from the top to the bottom of the measuring chamber and across it, as shown in Figure 7. The gas flow through sensor cells is presented schematically in Figure 7b–d. The specially designed organization of gas movement in the sensor’s cells and the small size of the cells allowed for a quick and complete exchange of the analyzed gas sample. Based on the fan airflow, the complete air exchange in the sensor chamber takes less than one second. As a result of this, the device was characterized by a very short response time regarding changes in the composition of the gas sampled by the fan.

#### 3.3.2. Sensing Module PMS5003

The sensing module PMS5003 is a commercial product. It includes sensors for temperature, formaldehyde, and particulate matter. Their measurement characteristics are shown in Table 1.

**Figure 8:**
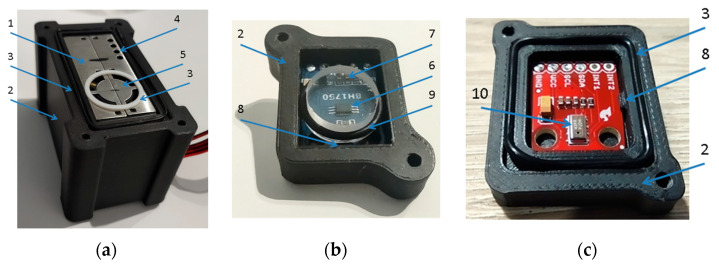
(**a**) PMS5003 sensing module inside PCV chamber. (**b**) BH1750 inside PCV chamber. (**c**) MPL3115A2 inside PCV chamber. 1—PMS5003, 2—PCV chamber, 3—O-ring sealing, 4—gas inlet, 5—gas outlet, 6—BH1750 sensor inside PCV chamber, 7—transparent plexiglass, 8—PCB holding clips, 9—O-ring sealing which protects plexiglass, and 10—MPL3115A2.

**Figure 9:**
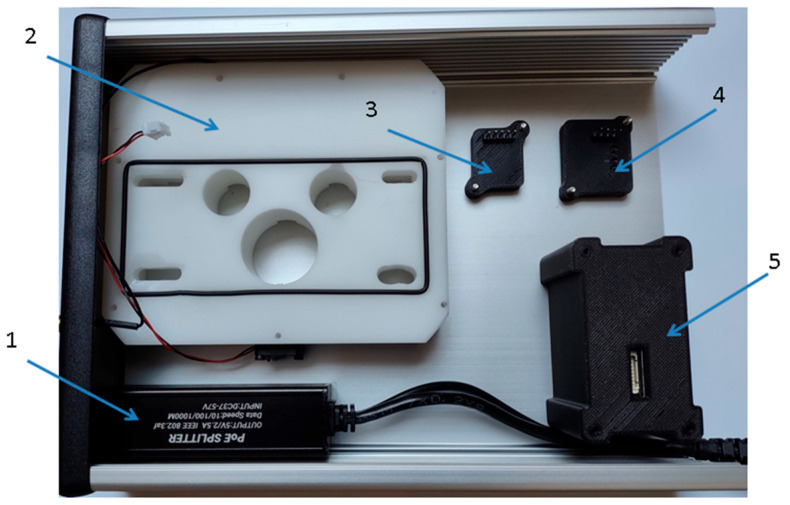
The arrangement of the individual modules inside the housing of the multisensor device (PCB was removed from the top of the measurement chamber so as not to overshadow the pressure sensor). 1—power supply PoE, 2—multi-cell sensing module, 3—BH1750 inside PCV chamber, 4—MPL3115A2 inside PCV chamber, and 5—PMS5003 sensing module inside PCV chamber.

PMS5003 is fitted with the active gas flow, which is enforced by the miniaturized in-built fan located at the outlet of the module. The maximum power consumption of the module is 100 mA. Two measures were undertaken to minimize the impact of the PMS5003 active gas flow on the other sensing modules installed inside the measurement device. First, the module was located in its own chamber, which perfectly fits the module and isolates it from the interior of the measurement device; see Figure 8a. The chamber was made of PCV using 3D technology. Second, the module was mounted to the device housing at a distance from the multi-cell sensing module and from the pressure sensor; see Figure 9.

#### 3.3.3. Sensors for the Measurement of Light Intensity and Pressure

The BH1750 sensor was chosen for visible light intensity measurement; see Table 1. The sensor was mounted in its own chamber, which was made of PCV using 3D technology; see Figure 8b. The entire module was screwed to the housing of the measurement device. Sensor exposure to the radiation was ensured using the plexiglass which was fitted on top of the sensor. Inside the measurement device, the module was located between the multi-cell sensing module and the pressure sensor; see Figure 9.

The atmospheric pressure is measured using the SEN-11084 module; see Table 1. The sensor was mounted in its own chamber, which was made of PCV using 3D technology; see Figure 7c. Its location inside the multisensor device is shown in Figure 9.

#### 3.3.4. Pneumatic Assembly

The dynamic sampling is performed by a suitable pneumatic system which withdraws the representative portion of indoor air and conveys it to the measurement chamber.

A small 5 V DC fan, size 17 mm × 17 mm × 8 mm (airflow 1.52 m^3^/h for a static pressure of 7.11 mm H_2_O), was mounted on one side of the measurement chamber; see Figure 7a,b. The fan maintains the gas flow and homogenous gas concentration inside the chamber. But it remains in contact only with the gas that leaves the measurement chamber through the outlet gas collector. The air, which is pulled out from the measurement chamber by the fan, gets to the free space inside the device housing (see Figure 1c), and ensures the efficient cooling of the electronic elements inside the measurement device. The fan speed is controlled in real time based on the settings provided by the device user. DAC was applied for fan control using the bipolar transistor BC817.

The maximum fan power consumption is 0.5 W. The gas chamber design which allows us to use the miniature fan for dynamic gas sampling instead of a vacuum pump allows for cutting the energy consumption of the pneumatic system by several dozen percent. Additionally, the device with the fan is quiet and may be used indoors while the devices fitted with the vacuum pump are not accepted by occupants.

#### 3.3.5. Filters

**Figure 10:**
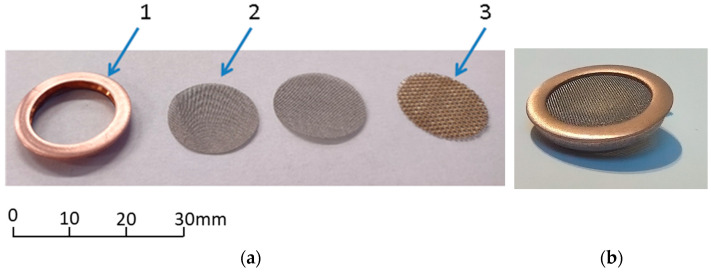
Filter construction. (**a**) Filter elements: 1—filter corps, 2—net, mesh size 0.04 mm, and 3—net, mesh size 0.2 mm. (**b**) Filter as an assembly of elements.

To prevent the measurement device damage resulting from the test gas contamination by coarse particles, all gas inlets of the individual sensing modules have been protected by filters; see Figure 1. An individual filter was composed of three layers of the stainless steel net, mesh size 0.2 mm and 0.04 mm, which are pressed into the copper corps, as shown in Figure 10. The filters are replaceable. They fit in the dedicated openings in the device housing which makes them easy to exchange. Such a solution is convenient. It does not generate a notable flow resistance while allowing for measurement system protection.

#### 3.3.6. Data Transmission and Interfaces

An interface is the coupling between a system that is being considered and another system, or between devices of a system, through which information passes. In the case of the presented instrument, remote control and data acquisition can be performed by an external personal computer through the standard communication UART port with a baud rate of 9600 bit/s or using a wireless WiFi network.

Stable data transmission, without delays and breaks, is crucial for the developed application. The wireless data transmission option was chosen as the primary solution. We used the microcontroller board with an inbuilt WiFi module and the external antenna, fitted with an SMA connector. The communication system may be easily extended by fitting the device with the GSM module, for GSM transmission, or by connecting the Ethernet module, for wired transmission, using the RJ45 cable and utilizing the PoE power supply technology. All these solutions allow for the transmission of measurement results.

The MQTT protocol was applied for data transmission, and device communication with other devices and with the user. This protocol is light and simple and it has a low throughput. The data are sent upon the query initiated by the user or paired external device. To attain communication, the MQTT broker receives messages from publishing clients, preceded by the adequate parameter ”TOPIC” which identifies the target device and then distributes them to the appropriate subscribing customers. The wireless communication interface allows the user to:

- Publish the current value of an individual sensor in the MQTT network;
- Publish files with the measurement data in the MQTT network;
- Publish the current configuration of the measurement device in the MQTT network;
- Publish the information about data transmission errors and sensing elements errors in the MQTT network;
- Subscribe the information from the auxiliary device and save it on the microSD card;
- Subscribe commands that change the current configuration of the network, devices, and sensors;
- Subscribe commands which allow us to modify files saved on the microSD card;
- Subscribe commands which manage the measurement device (switch on, switch off, reset, etc.).

**Figure 11:**
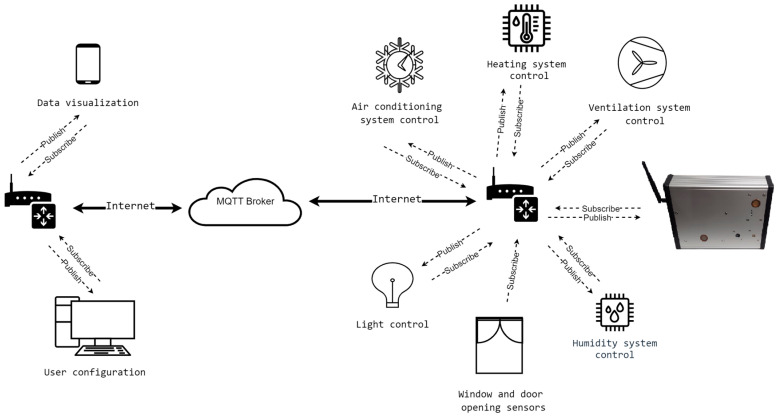
Multisensor device as IoT element for indoor environment monitoring.

The applied MQTT protocol allows the multisensor device to access the information from the auxiliary devices and record it on the internal microSD card. This option enables a centralized solution, where the main measurement device collects all the information about the indoor environment from its sensors, as well as from the auxiliary devices, so that it is available in one place. In such cases, the auxiliary devices may be energy-efficient and very simple in terms of construction. The MQTT protocol also allows the multisensor device to deliver information about the indoor environment to the controllers of the devices and system which are dedicated to improving indoor conditions (e.g., HVAC). As the device collects multiple kinds of data, its utility is very high. Figure 11 presents the multisensor device as an IoT element for indoor environment monitoring.

#### 3.3.7. Microcontroller

**Figure 12:**
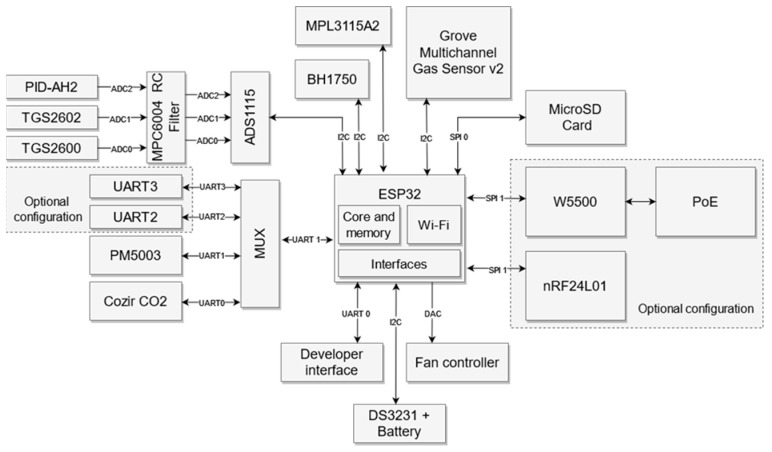
Block diagram of the measurement device and its operation control by EPS microcontroller.

The microcontroller ESP-WROOM-32 (ESPRESSIF, Shanghai, China) was chosen and applied to control the operation of the measurement device; see Figure 12. It has 520 kB SRAM memory, 8 MB FLASH memory, and a Dual Core Tensilica LX6 processor (Santa Clara, CA, USA) with a frequency of 240 MHz. The microcontroller is equipped with multiple, diverse communication interfaces and a wireless communication system, using a 2.4 GHz frequency.

The main communication interface of the EPS microcontroller is the I2C bus. It was used to transfer the information between the microcontroller and ADC converter, BH1750 (light intensity sensor), Grove Multichannel Gas Sensor v3, and MPL3115A2 (atmospheric pressure sensor). The ADC interface was equipped with the I2C bus voltage adjustment circuit, based on two Mosfet transistors. The logic levels converter ensures two-way communication over SDA and SCL lines between the ADC converter (operating voltage 5 V), and the digital part (operating voltage 3.3 V).

The Cozir-Blink5000 module and PMS5003 module communicate with the EPS microcontroller via the UART interface. Based on the characteristics of these devices, the measured value readout interval is 10 s, in both cases. This implies a small throughput of information readout for the EPS microcontroller. In such a case, it is sufficient to use a fast four-channel, double multiplexer, with low power consumption, as the UART interface extension. Such a solution allows us to use smaller and cheaper microcontroller, which has only one UART interface.

The measurement data are stored on the microSD card, which communicates with the EPS microcontroller via the SPI0 bus. Another serial interface SPI1 is utilized for communication with data transmission modulesDepending on the applied configuration, they may be the wireless communication systems RF or GSM, or the wired Ethernet system.

**Figure 13:**
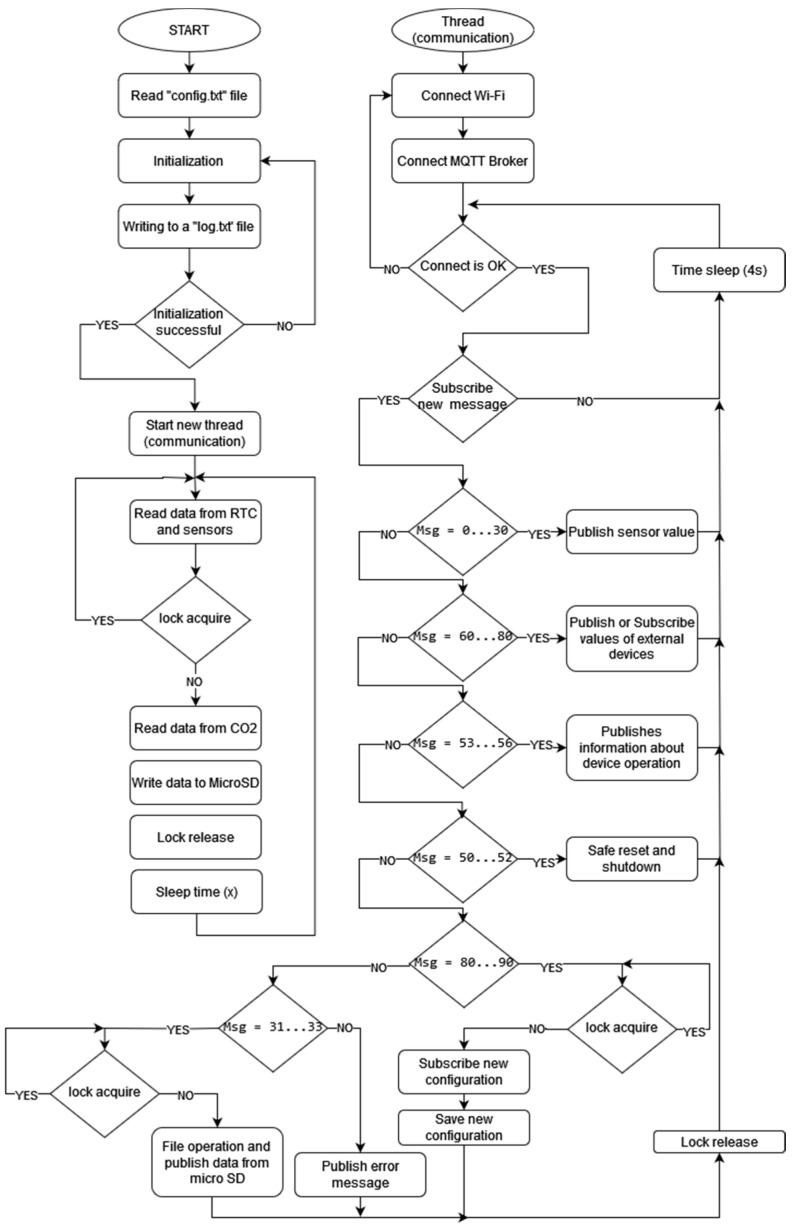
Working algorithm of EPS microcontroller.

The EPS microcontroller allows for multithreading. The working algorithm of the EPS microcontroller applied in the measurement device is shown in Figure 13. The first thread is responsible for the device initialization, sensor readout, data acquisition, and recording on the microSD card. The second thread deals with the communication interface. It interprets incoming requests and responds. Configuration files are saved on the microSD card. The files are available to the microcontroller for interpretation, to attain the proper configuration of the measurement device. Multithreading may generate access conflicts. To prevent requests for the simultaneous access of two threads to the same resources, a synchronizing element was introduced. This ensures the thread has exclusive access to the shared resource.

The software Thonny 4.0.2 using the MicroPython language was applied to program the ESP32 microcontroller.

The proper operation of the microcontroller is secured by the watchdog (WTD). To prevent the unexpected shut-down of the device, the WTD system will reset the microcontroller. The microcontroller reset will automatically initialize the system so that it is capable of data acquisition and publishing in the MQTT network.

#### 3.3.8. Power Supply

Given the request for the continuous and uninterrupted operation of the monitoring system, the reliability of the power supply is very important. The batteries were not considered as the major, or exclusive, means of power supply. It is assumed that a power outlet is directly available in the room and the sensor device can be directly plugged in.

**Figure 14:**
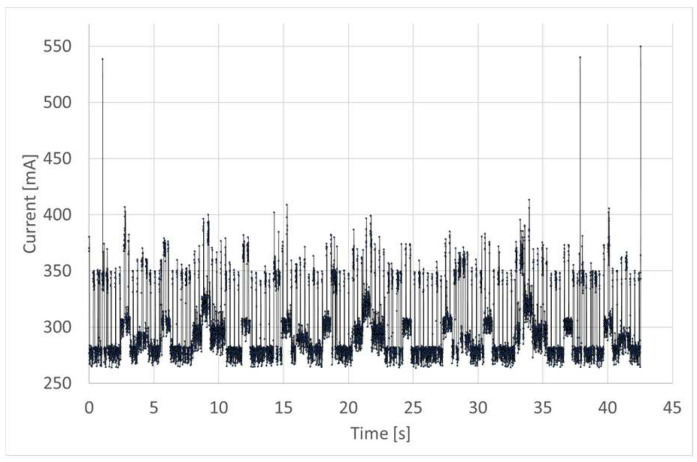
Device input current measured over the period including three measurement cycles.

The energy consumption upon normal device operation was determined based on measuring the input current. Figure 14 shows the record of the input current including three measurement cycles (one cycle lasts 10 s). Three 500 mA peaks represent active wireless transmission, when all measurement data are published by the device in the MQTT network and they are received by the user. This operation takes 1 ns. On average, the input current oscillated around 301 mA which corresponds to power consumption at the level of 1.5 W. Still, mains powering is required to attain the measurement resolution at the level of seconds and no restrictions on the frequency of contacting the cloud. Such requirements were imposed on the device, and the use of mains powering was a deliberate choice.

The device is mains-powered, fusing a power supply transformer 230 V AC to 5 V DC (3A current). A dedicated power supply module was implemented to eliminate disturbances in digital data processing. They could be caused by the interference from the electromagnetic field associated with the electrical engine and high-frequency wireless transmission in the device. The module utilizes a system of linear voltage stabilizers, capacitors, and voltage chokes (inductance 15 uH), which damp high-frequency disturbances. Each major module in the device has its separate power supply composed of an analog circuit with a voltage of 5 V, a digital circuit with a voltage of 3.3 V, and a sensing circuit with a voltage of 3.3 V.

#### 3.3.9. Instrument Housing

To ensure continuous and reliable operation, the components of the sensor device must be protected against the action of various environmental factors. They have to withstand humidity, dust, and various mechanical factors. At the same time, the device has to be relatively easy to carry and allocate for use.

The aluminum housing for electronics was applied (Hammond Manufacturing, Guelph, ON, Canada), size 220 mm × 165 mm × 51.5 mm. It is made of a pressed aluminum profile, which is closed by two side panels; see Figure 1. The housing has good electromagnetic compatibility (ECM) and an IP54 protection certificate. The arrangement of the individual modules of the multisensory device inside the housing is shown in Figure 14.

## 4. Exemplary Results of Indoor Environment Monitoring with the Multisensor Device

**Figure 15:**
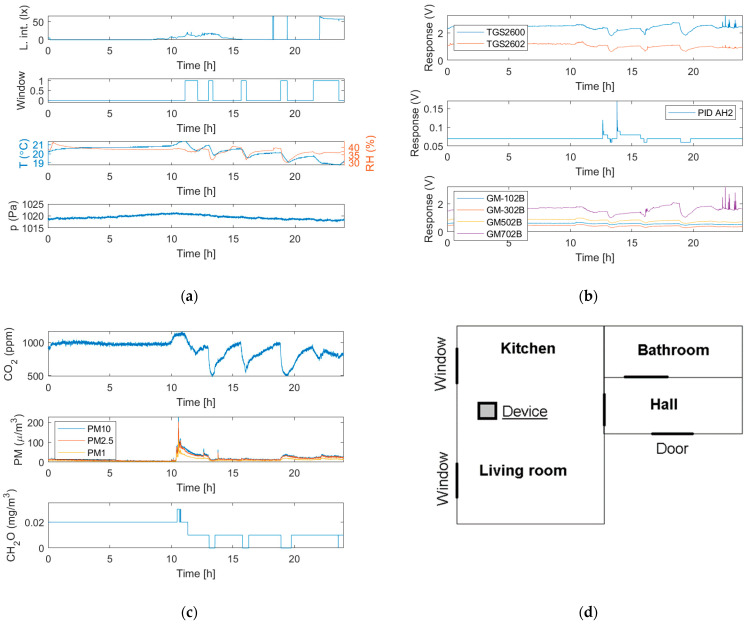
(**a**–**c**) Results of 24 h monitoring of the indoor environment with the multisensor device. (**d**) Apartment layout and measurement device location.

The multisensor device presented in this paper has been prototyped and it is fully operational. Figure 15 presents the results of indoor environment monitoring lasting 24 h. It was a part of the ongoing test measurement session, which is currently over 2 weeks long.

The monitoring was realized in the occupied apartment; see Figure 15d. The flat consisted of a dining room with an open kitchen (26 m^2^), bathroom (3.5 m^2^), and hall (3.1 m^2^). The device was located in the dining room, on the table (75 cm above the floor) at a distance of 2 m from the middle of the kitchen zone and 1.4 m from the windows.

Based on Figure 15a–c, the measurement results well reflected the conditions in the indoor space resulting from its use by occupants, as well as the impact of the outdoor environment. The top chart in Figure 15a indicates the periods when the kitchen was lit by daylight and when artificial light was used by the occupants, including the associated differences in light intensity. During measurements, the air temperature and humidity (Figure 15a, third chart) remained in the comfort range, although a higher humidity could be advantageous. Both parameters were strongly influenced by window opening and closing, as shown by the correlation with Figure 15a, second chart. Opening the window caused the temperature and relative humidity drop, which is typical in moderate climate zones during winter. It also greatly influenced the CO_2_ concentration (compare Figure 15a, second chart, and Figure 15c, top chart), causing its drop, as a result of weathering. When the window remained closed, the CO_2_ concentration approached 1000 ppm, and, also, at night, it remained at this level, while lower values would be recommended in general. Window opening was also reflected in the VOC content as indicated by the TGS sensors (Figure 15b, top chart) and GM sensors (Figure 15b, bottom chart). The PID sensor was not indicative in this respect (Figure 15b, middle chart). Based on the occupants’ observation, a high PM concentration peak at 10 a.m. was associated with vacuum cleaning. As shown, it faded away fairly slowly. Smaller peaks, which appeared later, could be correlated with window opening and PM intake with ambient air. Formaldehyde concentration changes were minor (Figure 15c, bottom chart). Indoor pressure seemed insensitive to kitchen use. As demonstrated, the multisensing device is a precious source of exhaustive information about the indoor environment.

## 5. Summary

A multisensor device featuring an innovative design and construction was presented in this work. It is a direct-reading instrument that can become an element of IoT. This portable device offers real-time measurements of a wide spectrum of physical and chemical quantities with online data access and visualization. It is compact, small, and light, and, hence, easy to move between places. The device has a relatively low power consumption. It operates automatically, can be operated unattended, and requires minimal intervention. Only periodic checks by a specialized service are required. The device can collect a huge amount of data about the indoor environment, to provide the information about it to the IoT. The device can be configured to control actuators of various devices and equipment including external systems used for ventilation, heating, and air conditioning.

## Figures and Tables

**Table 1:** Measurement characteristics of all sensors mounted in the device.

Sensors		Target	Measurement Accuracy	Measurement Range
MPL3115A2 (NXP Semiconductors, Eindhoven, The Netherlands)		Pressure	0.1 kPa	50 to 110 kPa
BH1750 (OKYSTAR, Shenzhen, China)		Light intensity	1 lx	1–65,535 lx
TGS2600 (Figaro Engineering Inc., Osaka, Japan)		Hydrogen	-	1–30 ppm
TGS2602 (Figaro Engineering Inc., Osaka, Japan)		Ethanol	-	1–30 ppm
PID AH-2 (Alphasense, Great Notley, UK)		Isobutylene	-	1 ppb–50 ppm
Cozir-Blink5000 (Gas Sensing Solutions Ltd., Cumbernauld, UK)		Carbon dioxide	30 ppm	0–5000 ppm
Grove Multichannel Gas Sensor V2 (Seeed Technology Co Ltd., Shenzhen, China)	GM-102B	Nitrogen dioxide	-	0.1–10 ppm
	GM-302B	Ethanol	-	1–500 ppm
	GM502B	Volatile organic compounds, ethanol	-	1–500 ppm
	GM702B	Carbon monoxide	-	5–5000 ppm
PMS5003 (DFRobot, Shanghai, China)		Temperature	0.1 °C	−20 °C–99 °C
		Humidity	0.1%	0–99%
		Formaldehyde	0.01 mg/m^3^	0–2 mg/m^3^
		Particulate matter (PM)	1 μg/m^3^	0–500 μg/m^3^

**Table 2:** Free volume available for the test gas inside the measurement chamber.

Compartment For	Free Volume without Sensor [cm^3^]	Free Volume with Sensor Inside [cm^3^]
TGS2600	7.3	5.6
TGS2602	7.3	5.6
PID AH-2	14.3	5.8
Grove Multichannel Gas Sensor V2	9.7	5.7
Cozir-Blink5000	11.8	5.5
Inlet channels including inlet collector	22.9	
Outlet channels including outlet collector	21.6	

## Data Availability

All data have been included in the paper.

## References

[1] Oh D., Kim J., Kim H., Jang H., Hong T., An J. (2023). Analyzing the impact of indoor environmental quality on physiological responses and work performance: Implications for IEQ control strategies. Build. Environ..

[2] Khalid N.S., Abdullah Y.A., Nasrudin N. (2022). How does the indoor environment affect mental health when working remotely. Plan. Malays. J..

[3] Felgueiras F., Mourao Z., Moreira A., Gabriel M.F. (2023). Indoor environmental quality in offices and risk of health and productivity complaints at work: A literature review. J. Hazard. Mater. Adv..

[4] Diaz M., Piderit M.B., Attia S. (2021). Parameters and indicators used in Indoor Environmental Quality (IEQ) studies: A review. J. Phys..

[5] Troncoso-Pastoriza F., Martínez-Comesana M., Ogando-Martínez A., Lopez-Gomez J., Eguía-Oller P., Febrero-Garrido L. (2022). IoT-based platform for automated IEQ spatio-temporal analysis in buildings using machine learning techniques. Autom. Constr..

[6] Roa C.D., Schiavon S., Parkinson T. (2020). Targeted occupant surveys: A novel method to effectively relate occupant feedback with environmental conditions. Build. Environ..

[7] Berquist J., Ouf M.M., O’Brien W. (2019). A method to conduct longitudinal studies on indoor environmental quality and perceived occupant comfort. Build. Environ..

[8] Saini J., Dutta M., Marques G. (2020). A comprehensive review on indoor air quality monitoring systems for enhanced public health. Sustain. Environ. Res..

[9] Mujan I., Licina D., Kljajic M., Culic A., Anđelkovic A.S. (2021). Development of indoor environmental quality index using a low-cost monitoring platform. J. Clean. Prod..

[10] Tiele A., Esfahani S., Covington J. (2018). Design and Development of a Low-Cost, Portable Monitoring Device for Indoor Environment Quality. Hindawi J. Sens..

[11] Saini J., Dutta M., Marques G. (2021). Sensors for indoor air quality monitoring and assessment through internet of things: A systematic review. Environ. Monit. Assess..

[12] Parkinson T., Parkinson A., de Dear R. (2019). Continuous IEQ monitoring system: Context and development. Build. Environ..

[13] Yang C.T., Chen S.T., Den W., Wang Y.T., Kristiani E. (2019). Implementation of an Intelligent Indoor Environmental Monitoring and management system in cloud. Future Gener. Comput. Syst..

[14] Jin M., Liu S., Schiavon S., Spanos C. (2018). Automated mobile sensing: Towards high-granularity agile indoor environmental quality monitoring. Build. Environ..

[15] Demanega I., Mujan I., Singer B.C., Anđelkovic A.S., Babich F., Licina D. (2021). Performance assessment of low-cost environmental monitors and single sensors under variable indoor air quality and thermal conditions. Build. Environ..

